# “Cyclic nucleotides in plants: from obscure messengers to central regulators“

**DOI:** 10.3389/fpls.2025.1618243

**Published:** 2025-06-25

**Authors:** Brygida Świeżawska-Boniecka, Adriana Szmidt-Jaworska

**Affiliations:** Department of Plant Physiology and Biotechnology, Faculty of Biological and Veterinary Sciences, Nicolaus Copernicus University in Toruń, Toruń, Poland

**Keywords:** cyclic AMP, cyclic GMP, adenylyl cyclase, guanylyl cyclase, plant signaling

## Abstract

This review examines the historical trajectory of cyclic nucleotides (cNMPs) research in plants, a field that has encountered prolonged challenges and skepticism, contrasting sharply with its rapid validation in animal systems. For decades, efforts to detect cyclic nucleotides, specifically cyclic adenosine monophosphate (cAMP) and cyclic guanosine monophosphate (cGMP), in plant tissues and to elucidate their functional roles were inconclusive. These challenges primarily stemmed from the extremely low endogenous concentrations of cNMPs in plant cells, the rapid turnover of these molecules, and the limited sensitivity and specificity of early analytical techniques. However, since the 1970s, significant advances in analytical methodologies and bioinformatics have enabled precise quantification of cNMP levels and the bioinformatics identification of enzymes central to plant cNMP signaling pathways. In this review, we trace the key milestones and transformative discoveries that have shaped the evolving landscape of cyclic nucleotide research in plants, highlighting how each step forward has deepened our understanding of cAMP and cGMP as integral regulators of plant physiology.

## Introduction

Knowledge about cyclic nucleotides such as cyclic adenosine 3’, 5’ monophosphate and cyclic guanosine 3’, 5’ monophosphate (cAMP and cGMP, respectively) in animals has advanced remarkably since the 1957 discovery that cyclic AMP functions as a second messenger in hormone action, such as that of adrenaline (Sutherland and Rall, 1957). These two simple signaling molecules have become fundamental to understanding animal regulatory systems and have contributed to five Nobel Prizes awarded to pioneering scientists in this field (Beavo and Brunton, 2002).

Unlike the rapid acceptance of cyclic nucleotides in animal research, recognizing their existence and importance in plant tissues has been a prolonged and challenging endeavor. For more than fifty years, efforts to detect cAMP or cGMP in plants often yielded inconclusive results or stirred debate, largely due to limitations in technology and methodology. This review retraces the journey from the 1970s to the present, highlighting the challenges, advancements, and breakthroughs in accurately measuring cNMP levels in plant tissues, as well as in the bioinformatics identification and characterization of enzymes involved in cNMP signaling pathways. We also examine the key milestones in plant cyclic nucleotide research, showcasing how the development of analytical and bioinformatics tools has led to major discoveries in the field.

Several researchers have suggested that cyclic nucleotide signaling operates through analogous mechanisms across a diverse array of life forms, including bacteria, fungi, plants, and animals, involving similar enzymes and downstream effectors such as protein kinases and ion channels. However, in plant systems, the situation proved to be more intricate and complex, and it was not merely a matter of replicating the signaling pathways observed in animals and prokaryotes. As a result, the existence and functional significance of cyclic nucleotides in plants remained a topic of vigorous debate for nearly three decades, beginning in the late 1960s.

One significant reason for the hesitant acceptance of these signaling molecules in higher plants was the observation that cAMP and cGMP levels in plants are generally lower than those found in animals or lower eukaryotes. One of the overlooked limitations of earlier studies on plant cNMPs is the lack of consideration for their time- and tissue-specific localization (e.g. pollen tube, aleurone layer) (Moutinho et al., 2001; Penson et al., 1996). Due to the use of large, blended tissue samples and low-sensitivity analytical methods, localized accumulation of cAMP or cGMP in specific cell types or plant developmental stages could have remained undetected. Additionally, early research on plant cyclic nucleotide signal transduction was heavily focused on identifying genetic and molecular similarities to cNMP mechanisms observed in animals and bacteria. This lack of significant homology proved frustrating for scientists, as it was well-established that cyclic nucleotides are present in plant tissues (Cousson and Vavasseur, 1998; Isner et al., 2012; Jin and Wu, 1999; Penson et al., 1996). Nonetheless, the proteins responsible for their synthesis, deactivation, and signal transduction remained elusive, complicating the understanding of cNMP signaling in plants.

**Figure 1:**
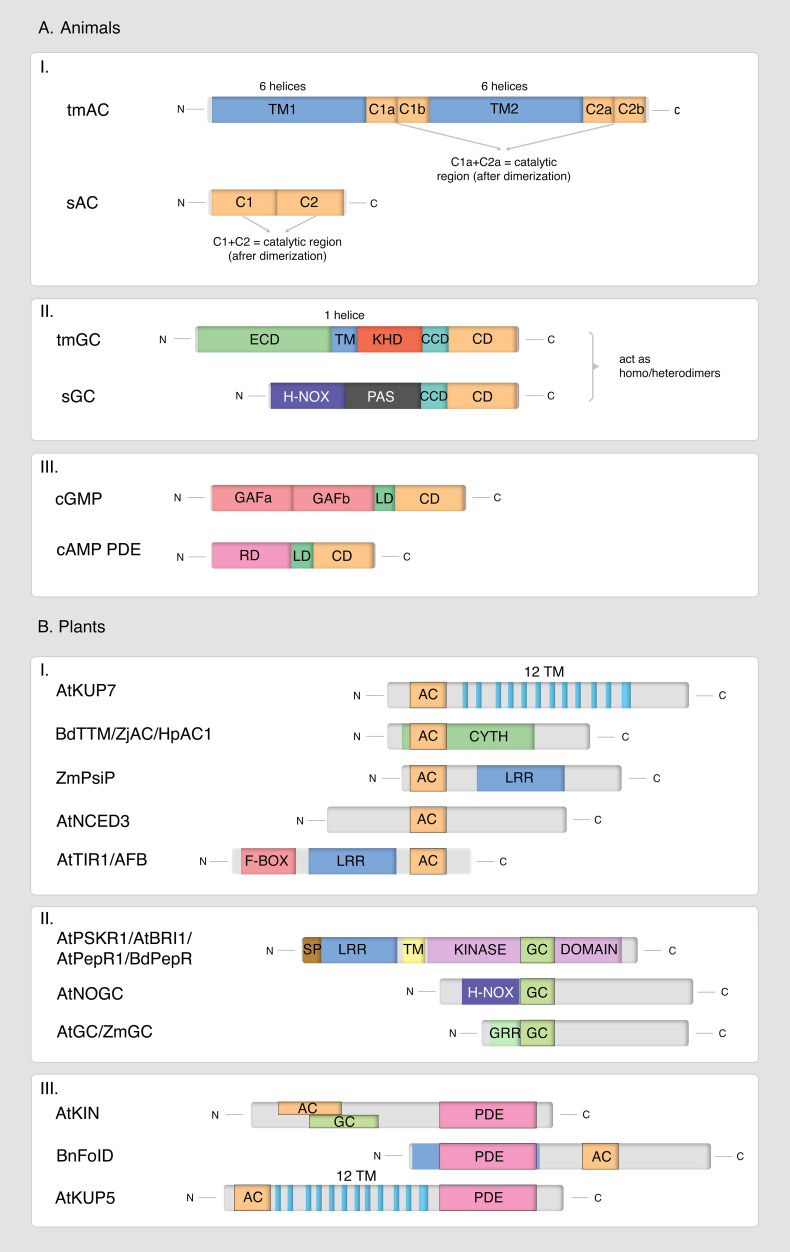
Divergent domain architecture of adenylyl cyclases (I), guanylyl cyclases (II), and phosphodiesterases (III) in animals **(A)** and plants **(B)**. **(A)** I. Domain organization of animal adenylyl cyclases. tmAC – transmembrane adenylyl cyclase; sAC – soluble adenylyl cyclase; TM1, TM2 – transmembrane domains; C1, C2 – catalytic domains. II. Domain organization of animal guanylyl cyclases. tmGC – transmembrane guanylyl cyclase; sGC – soluble guanylyl cyclase; ECD – extracellular domain; TM – transmembrane domain; KHD – kinase homology domain; CCD – coiled-coil domain; CD – catalytic domain; H-NOX – heme-nitric oxide/oxygen binding domain; PAS – Per-Arnt-Sim domain. III. Domain organization of animal phosphodiesterases. cGMP PDE – cGMP-specific phosphodiesterase; cAMP PDE – cAMP-specific phosphodiesterase; GAFa, GAFb – domains found in cGMP-regulated PDEs, Anabaena adenylyl cyclases, and *E* coli FhlA; LD – linker domain; CD – catalytic domain; RD – regulatory domain. **(B)** I. Domain organization of selected plant adenylyl cyclases showing diverse localization of the 14-amino acid adenylyl cyclase (AC) motif. AtKUP7 – *Arabidopsis thaliana* K⁺-uptake permease 7; BdTTM – *Brachypodium distachyon* triphosphate tunnel metalloenzyme; ZjAC – *Ziziphus jujuba* adenylyl cyclase; HpAC1 – *Hippeastrum hybridum* adenylyl cyclase 1; ZmPsiP – *Zea mays* pollen signaling protein; AtNCED3 – *A thaliana* 9-cis-epoxycarotenoid dioxygenase; AtTIR1/AFB – *A thaliana* transport inhibitor response1/auxin signaling F-box receptor; AC – adenylyl cyclase motif [RK][YFW][DE][VIL][FV]X(8)[KR]; TM – transmembrane domain; CYTH – catalytic domain of thiamine triphosphatase and CyaB-like adenylyl cyclase; LRR – leucine-rich repeat domain; F-box – F-box domain. II. Domain organization of selected plant guanylyl cyclases with diverse localization of the 14-amino acid guanylyl cyclase (GC) motif. AtPSKR1 – *A thaliana* phytosulfokine receptor 1; AtBRI1 – *A thaliana* brassinosteroid receptor; AtPepR1 – *A thaliana* peptide signaling receptor; BdPepR2 – *B distachyon* peptide signaling receptor 2; AtNOGC1 – *A thaliana* flavin monooxygenase; AtGC1 – *A thaliana* guanylyl cyclase 1; ZmGC – *Z. mays* guanylyl cyclase; GC – guanylyl cyclase motif [RKS][YFW][CTGH][VIL][FV]G[DNA]X[VIL]X{4}[KR][RKS]; SP – signal peptide; LRR – leucine-rich repeat domain; H-NOX – heme-nitric oxide/oxygen binding domain; GRR – glycine-rich region. III. Domain organization of selected plant phosphodiesterases, illustrating diverse localization of the phosphodiesterase (PDE) search motif. AtKIN – *A. thaliana* subunit of SnRK2 kinase exhibiting AC, GC, and PDE activities; BnFolD – *Brassica napus* methylenetetrahydrofolate dehydrogenase; AtKUP5 – *A. thaliana* K⁺-uptake permease 5; PDE – phosphodiesterase motif [YFW]Hx[YFW]Rx{20,40}[HRK][DE]; AC – adenylyl cyclase motif; GC – guanylyl cyclase motif; TM – transmembrane domain.

At present, advancements in highly sensitive detection methods, such as liquid chromatography tandem mass spectrometry (LC-MS/MS), along with the utilization of catalytic center motif searches, recombinant technologies, and omics approaches, have facilitated the discovery and characterization of numerous components involved in cNMPs signaling in plants (Qi et al., 2010; Al-Younis et al., 2015, 2018, 2021; Bianchet et al., 2019; Świeżawska et al., 2017, 2020; Liu et al., 2023; Yuan et al., 2022, 2023; Kwiatkowski et al., 2021a, 2021b). Key enzymes such as adenylyl cyclases (ACs), guanylyl cyclases (GCs), and phosphodiesterases (PDEs), which are responsible for the synthesis and hydrolysis of cAMP and cGMP, respectively, have been identified as central players in this signaling cascade. Recent studies have revealed that plant enzymes exhibit distinct structural and regulatory features compared to their animal counterparts (Sunahara and Taussig, 2002; Visweswariah and Jaiswal, 2016; Wong et al., 2023). Unlike the distinct, isolated proteins typically found in animals, plant ACs, GCs, and PDEs are often integrated as individual domains within larger, multifunctional proteins (**Figure 1**). This finding underscores the complexity of plant cNMP signaling systems. It has become evident that previous efforts to identify plant enzymes by drawing parallels to well-characterized animal proteins have proven largely ineffective. Rather than functioning as isolated proteins with clearly defined, organized domains, these plant enzymes operate as “moonlighting proteins,” possessing short catalytic centers for AC, GC or PDE activity embedded within multifunctional proteins that serve multiple biochemical and/or biological roles (Wong et al., 2018).

Terms like “moonlighting proteins,” “crypto-AC/GC”, “hidden AC/GC” and “twin AC/GC-PDE proteins” have become commonplace in discussions regarding plant cNMP cyclases and phosphodiesterases (Irving et al., 2025). These designations underscore the notion that the catalytic centers of these enzymes are embedded within multifunctional proteins (Wong et al., 2020, 2023; Kwiatkowski et al., 2021a; Turek and Irving, 2021; Turek et al., 2023).

## Periods of uncertainty and areas of speculation

The roles of cyclic nucleotides in cellular and biological processes in higher plants are now widely recognized and extensively documented in contemporary literature (Lemtiri-Chlieh et al., 2011; Blanco et al., 2020; Wong et al., 2023, Świeżawska-Boniecka and Szmidt-Jaworska, 2023; Figueroa et al., 2023; Qi and Friml, 2023). However, early studies published in the late 1960s and 1970s, which suggested the presence and significant roles of cAMP and cGMP in the physiological processes of higher plants, faced substantial criticism (Azhar and Murti, 1971; Brown and Newton, 1973; Janistyn, 1972; Salomon and Mascarenhas, 1972). This skepticism arose primarily from several key issues: 1/sensitivity limitations - the analytical methods employed at the time lacked the sensitivity needed to measure cNMP concentrations at the picomole or femtomole level; 2/specificity concerns - many of the early assay techniques were unable to differentiate between isomers, notably failing to distinguish 2’:3’-cAMP from the more relevant isomer, 3’:5’-cAMP; 3/purification challenges - the need for complex purification techniques prior to assay further complicated the measurement process (Brown and Newton, 1981; Amrhein, 1974; Keates, 1973; Lemtiri-Chlieh et al., 2011). Skeptical papers with titles such as “Evidence Against the Occurrence of Adenosine 3’:5’-Cyclic Monophosphate in Higher Plants” (Amrhein, 1974) and “Evidence That Cyclic-AMP Does Not Mediate the Action of Gibberellic Acid” (Keates, 1973) highlighted numerous methodological flaws, particularly concerning the inadequate chromatographic identification of cNMPs.

It has become evident that the purification and detection techniques for cyclic nucleotides, originally developed for animal tissues, were unsuitable for plant systems due to significant differences in cNMP concentration levels and enzyme structures between these kingdoms (**Figure 1**). Once the controversies surrounding the presence of cAMP and cGMP in plants were resolved through the advancement of measurement techniques, attention shifted to identifying the enzymes involved in cNMP signaling pathways. A landmark study published in 1997 in *Nature* introduced a potential plant adenylyl cyclase, named Axi141, discovered in tobacco (Ichikawa et al., 1998).

Sequence analysis indicates that the tobacco adenylyl cyclase is likely a soluble protein that features characteristic leucine-rich repeats and shows similarity to the adenylyl cyclase found in the yeast *Schizosaccharomyces pombe*. Biochemical assays utilizing the Biotrack immunoassay method confirmed the biosynthesis of cyclic AMP by Axi141. Furthermore, the authors suggested a strong correlation between the presence of cAMP and the auxin signaling pathway in tobacco protoplasts. However, this paper has since been retracted, with the authors stating: “the data showing that cAMP can stimulate protoplast division in the absence of auxins are not correct.” Unfortunately, there are no additional results regarding the adenylyl cyclase activity of the Axi141 protein, leaving its functionality in question. Consequently, the first plant protein suggested to possess adenylyl cyclase activity was the pollen signaling protein (PsiP) from Zea mays, which played a crucial role in cAMP-dependent polarized pollen tube growth (Moutinho et al., 2001). Meanwhile, the first identified plant guanylyl cyclase, AtGC1 from *Arabidopsis thaliana*, was discovered two years later (Ludidi & Gehring, 2003).

In subsequent years, numerous studies demonstrated that both adenylyl cyclase and guanylyl cyclase activities are often detected as ‘additional’ functions in multi-domain complex proteins, referred to as ‘crypto-AC/GC’ or ‘moonlighting AC/GC’ (Kwezi et al., 2007, 2011; Meier et al., 2010; Qi et al., 2010; Al-Younis et al., 2015, 2018, 2021; Bianchet et al., 2019; Świeżawska et al., 2017, 2020; Liu et al., 2023; Yuan et al., 2022, 2023; Kwiatkowski et al., 2021a, 2021b). Despite substantial evidence supporting the existence of cAMP/cGMP signaling systems in plant tissues, skepticism persists within the scientific community. For instance, the guanylyl cyclase activity of the brassinosteroid receptor AtBRI1 from *Arabidopsis thaliana*, as reported by Kwezi et al. (2007) using the cGMP enzyme immunoassay Biotrak (EIA) System, was not confirmed in later studies analyzing its crystal structure (Bojar et al., 2014). Additionally, this activity was measured using high-performance liquid chromatography (HPLC), which may not be sensitive enough to detect the low levels of cGMP produced by plant GCs (expressed in femtomoles per microgram of protein).

The guanylyl cyclase activity of another moonlighting protein, AtPepR1, from *Arabidopsis thaliana* (Qi et al., 2010) has also been met with significant skepticism, as highlighted in a letter titled “Guanylyl cyclase activity in plants?” (Ashton, 2011). The author criticized the “extraordinarily low” activity of AtPepR1 and other identified plant guanylyl cyclases in comparison to their animal counterparts, questioning the biological relevance of such enzymes in plants. Ashton suggested that the observed levels of cGMP could be artifacts or the result of bacterial contamination. Furthermore, author emphasized the need to produce and biochemically characterize the full-length recombinant AtPepR1 protein, rather than focusing solely on the fragment containing the guanylyl cyclase domain. Berkowitz et al. (2011) swiftly addressed Ashton’s theoretical speculations, thoroughly discussing all doubts raised. This engaging exchange in the PNAS journal’s forum highlighted that, even 30 years after the identification of cyclic nucleotides in plants, the topic remains controversial.

Similar doubts have been raised regarding plant adenylyl cyclases. The first experimentally confirmed adenylyl cyclase from the CYTH domain-containing family was HpAC1 from *Hippeastrum hybridum* (Świeżawska et al., 2014). The AC activity of HpAC1 was validated using two independent methods: (^3^H) cAMP radioimmunoassay (RIA) kit and complementation of the *cyaA* mutation in *E. coli* with the *HpAC1* gene. Subsequent studies demonstrated that several orthologs of HpAC1 in other plant species, such as *Brachypodium distachyon*, *Malus domestica*, *Ziziphus jujuba*, and *Pyrus × bretschneideri* may also function as adenylyl cyclases (Świeżawska et al., 2020; Yuan et al., 2022, 2023; Liu et al., 2023). However, it is important to note that the AC activity of these enzymes appears to be secondary, as they are primarily characterized by other dominant functions, such as significant triphosphatase activity. In 2020, Kleinboelting et al. published findings indicating that their enzymatic characterization of HpAC1 revealed a lack of detectable adenylyl cyclase activity using UHPLC and highlighted its predominant triphosphatase activity (Kleinboelting et al., 2020). They proposed renaming HpAC1 to HpPP1 (polyphosphatase 1) or HpTTM1 (triphosphate tunnel metalloenzyme 1), reflecting its primary function. The authors asserted that there is currently no plant enzyme with confirmed significant adenylyl cyclase activity, and that no plant proteins clearly acting as cAMP-binding proteins have been identified. As a result, the existence of cAMP signaling systems in higher plants remains unproven. This statement calls into question the validity of previously reported plant proteins with adenylate cyclase activity (Kasahara et al., 2016; Al-Younis et al., 2015, 2018; Chatukuta et al., 2018; Bianchet et al., 2019; Ruzvidzo et al., 2019). In response to the claims made by Kleinboelting et al., unpublished data have demonstrated the adenylyl cyclase activity of HpAC1 using a highly specific and sensitive technique such as liquid chromatography with tandem mass spectrometry. The results revealed that HpAC1 is an active adenylyl cyclase, producing approximately 18 fmol of cAMP per µg of protein per minute. This confirmation has been submitted to the journal’s editor as a letter.

Controversies surrounding plant cyclic nucleotide signaling frequently arise from inadequate measurement techniques and an excessive focus on drawing parallels between plant and animal cNMP signaling mechanisms. Historically, the analysis of plant systems has often been conducted solely through the lens of animal models, which is a perspective that is now considered outdated. This approach fails to acknowledge that plants and microorganisms were among the earliest organisms to inhabit the Earth, suggesting a rich evolutionary history that has led to the development of unique signaling pathways.

While it is true that both plants and animals exhibit functional similarities in their signaling processes, it is crucial to recognize that the underlying mechanisms and structural components are often markedly different. For instance, the enzymes and receptors involved in cNMP signaling may vary significantly between these groups, resulting in distinct regulatory pathways. These differences can lead to unique responses to environmental stimuli, growth signals, and developmental cues that are essential for the survival and adaptation of each organism.

Moreover, the reliance on measurement techniques developed for animal systems can obscure the true nature of plant signaling pathways. Advances in technology and methodology are necessary to accurately capture the dynamics of cNMP signaling in plants, which may involve lower concentrations of cyclic nucleotides and different regulatory proteins than those found in animal systems. A more nuanced understanding of plant cyclic nucleotide signaling will require researchers to embrace the distinct biological contexts of plants and microorganisms, allowing for the exploration of novel mechanisms that have evolved in these organisms. By shifting the focus from a comparative framework to one that values the inherent complexity of plant biology, researchers can gain deeper insights into the unique roles of cyclic nucleotides in plant signaling and physiology.

## Advancements in analytical techniques for cNMP detection

As mentioned in the previous chapter, standard chromatographic methods for assessing cyclic nucleotide levels in plants faced significant limitations in their detection capabilities (Azhar and Murti, 1971; Brown and Newton, 1973; Janistyn, 1972; Salomon & Masharencas, 1971; Amrhein, 1974; Keates, 1973). An important advancement in the measurement of cNMP levels in plant extracts was published in 1981 by Johnson et al., which marked a significant update in the methodologies used for this purpose (Johnson et al., 1981). A highly specific and sensitive gas chromatography-mass spectrometry (GC-MS) assay enabled the measurement of endogenous cAMP levels in various plant tissues, including tobacco (*Nicotiana tabacum* L.) callus cultures, bean (*Phaseolus vulgaris* L.) seedlings, and immature kernels of sweet corn (*Zea mays* L.), with concentrations ranging from 70 to 126 pmol/g of fresh weight. The authors also enhanced purification methods to obtain highly purified plant extracts for accurate quantification of cAMP. This included the removal of interfering substances such as scopolin, which was present in large quantities in callus tissue and exhibited similar chromatographic behavior to cAMP, thereby skewing the final results. Additionally, Johnson et al. developed a more efficient method for separating cAMP from its related isomer, 2′:3′-cAMP, addressing a specific challenge faced by earlier assay techniques (Brown and Newton, 1981). The detection limits for cNMPs in plant tissues remained insufficient. In 1989, Spiteri et al. published a study using radioimmunoassay (RIA) techniques, which found that after eliminating artefactual sources of cAMP and potential interfering substances, cAMP levels in five tested higher plant tissues (*Lactuca sativa*, *Helianthus annuus*, *Oryza sativa*, *Pinus pinaster*, and *Nicotiana tabacum*) were below the detection limit of 0.5 pmol/g of fresh tissue (Spiteri et al., 1989). This finding did not support the role of cAMP-dependent regulation in higher plants at that time; however, the RIA assay technique has since become one of the standard methods for cNMP assay in plants (Assmann, 1995). Other research groups utilizing the same RIA technique have reported significantly higher cAMP levels in plant extracts. For instance, concentrations of 36 pmol cAMP/g of fresh weight were detected in *Torenia* stem segments (Ishioka and Tanimoto, 1990), and 5 pmol cAMP/g of fresh weight were found in suspension-cultured cells of *Phaseolus vulgaris* (Bolwell, 1992). Radioimmunoassay is a relatively simple, sensitive, and specific method for measuring cyclic nucleotides in both animal and plant materials. Commercial sources, such as GE Healthcare, have developed RIA kits that allow for the convenient and rapid measurement of cAMP and cGMP (Gilman, 1970; Brooker, 1982; Harper and Brooker, 1975). The assay operates on the principle of competition between unlabeled cAMP/cGMP and a fixed quantity of ^125^I- or ^3^H-labelled cAMP/cGMP for a limited number of binding sites on a specific antibody. Typically, these kits provide two standard ranges: a less sensitive method suitable for liquid samples with high cAMP/cGMP levels (25–1600 fmol per tube) and a highly sensitive method, which includes an additional acetylation step for plasma and plant extracts (2–128 fmol per tube). The kits also ensure low cross-reactivity with other nucleotides. In studies, the biochemical activity of both adenylyl cyclase and guanylyl cyclase from *Hippeastrum hybridum* was measured using [^3^H] cAMP/cGMP RIA kits, and the resulting radioactivity was quantified with a scintillation counter (Świeżawska et al., 2014, 2015).

In recent years, enzyme immunoassay (EIA) has become the preferred non-radioactive method for measuring cNMPs, gradually replacing older radioactive techniques. Many companies now offer commercial cAMP and cGMP EIA kits that can detect very low levels of cNMPs in a variety of samples. These kits are especially suitable for plant samples, as they include an acetylation option that significantly increases sensitivity (to a range of about 2–128 fmol cNMP per well). EIA kits offers several benefits: high sensitivity (especially with the acetylation step), low interference from other nucleotides, and a quick and simple process. Due to these advantages, EIA remains a popular choice for measuring adenylyl and guanylyl cyclase activity in plants (Qi et al., 2010; Kwezi et al., 2011; Vaz Dias et al., 2019; Yuan et al., 2022, 2023; Świeżawska et al., 2020; Rahman et al., 2020; Dikobe et al., 2024). However, it is increasingly used alongside liquid chromatography-mass spectrometry, which has become a standard method for precise cNMP quantification in plant research.

In nucleotide chemistry, the integration of liquid chromatography with electrospray tandem mass spectrometry marked a significant leap forward in the precise identification and quantification of cyclic nucleotides in plant material, providing exceptional sensitivity (Witters et al., 1996, 1999). In 1996, Witters and colleagues highlighted that ion suppression HPLC coupled with MS/MS allows for routine cyclic nucleotide analysis by employing multiple reaction monitoring (MRM). This approach enables parallel quantification of various cyclic nucleotides at the femtomole level, a sensitivity previously only achievable through RIA or ELISA, though without the specificity that LC-MS provides (Witters et al., 1996). Recent advancements in chromatographic methods have established femtomole-level detection limits for cyclic nucleotides, fulfilling a longstanding need in plant cNMP research. Today, LC-MS/MS stands as the standard method for measuring cyclic nucleotides, including both 3’,5’-cNMPs and 2’,3’-cNMPs, as well as other nucleotide forms, with unparalleled sensitivity and specificity. Comprehensive workflows for nucleotide, nucleoside, and nucleobase analysis in plant samples have been further refined and reviewed in detail by Straube and colleagues, underscoring major progress in this field (Straube et al., 2021).

In recent years, LC-MS/MS has been successfully employed for several applications: (1) assessing adenylyl and guanylyl cyclase activity (Al-Younis et al., 2015, 2018, 2021; Bianchet et al., 2019; Świeżawska et al., 2020; Duszyn et al., 2021; Qi et al., 2022; Kwiatkowski et al., 2024), (2) conducting phosphodiesterase activity assays (Kwiatkowski et al., 2021a, 2021b; 2024), and (3) measuring total cNMP content in plant extracts (Qi et al., 2022; Chen et al., 2025).

Additionally, two innovative tools for detecting cyclic AMP, which complement classical analytical methods, have been developed. The first, known as the “cAMP sponge” (cAS) is a unique genetically encoded construct designed to elucidate the specific biological functions of cAMP within individual living cells. This tool provides valuable insights into the significance of compartmentalized signaling events (Lefkimmiatis et al., 2009). The “cAMP sponge” utilizes tandem cAMP-binding domains derived from the isolated regulatory subunit RIβ of protein kinase A (PKA-RIβ). It has been confirmed to effectively bind cAMP *in vitro* while remaining insensitive to cGMP. Additionally, the construct is tagged with the fluorescent protein mCherry, which is compatible with FRET-based sensors utilizing CFP and YFP.

The specific binding of cAMP to the sponge sequesters cAMP, leading to a reduction in its intracellular levels. This construct has been successfully utilized in tobacco Bright Yellow-2 (BY-2) cells, where it was shown that cAMP deficiency adversely affects cell growth, indicating that it is perceived as a stress condition by these cells (Sabetta et al., 2016). Following this, transgenic lines of *Arabidopsis thaliana* were created to overexpress the cAMP sponge (Sabetta et al., 2019). The authors reported that transgenic cAS plants, when buffered with cAMP and infected with an avirulent strain of *Pseudomonas syringae* pv. *tomato*, exhibited higher bacterial growth and reduced hypersensitive cell death compared to wild-type plants.

Another alternative tool for studying cyclic AMP is the complementation test of the *cyaA* mutation in *E. coli* adenylyl cyclase deficient strains, specifically the commercially available strains *E. coli* SP850 and CA8306 (Shah and Peterkofsky, 1991). This method provides a fast, straightforward and effective screening tool for evaluating candidates for adenylyl cyclase nomenclature. In this assay, the mutant *E. coli* strains, which lack the internal bacterial AC due to the *cyaA* mutation, are unable to induce expression of the lactose operon or utilize lactose. As a result, they produce colorless colonies on MacConkey agar. However, when these *cyaA*-deficient mutants are transformed with constructs containing an active AC insert, they can ferment lactose, leading to the formation of intensive red-colored colonies, indicating acid production. The complementation screening test was first utilized in 2001 by Moutinho and colleagues to confirm the AC activity of PsiP from maize (Moutinho et al., 2001). Since then, this valuable tool has been employed to verify the enzymatic activity of several potential plant ACs (Świeżawska et al., 2014, 2020; Al-Younis et al., 2015, 2018, 2021; Chatukuta et al., 2018; Yang et al., 2021; Yuan et al., 2022, 2023).

The review of current cyclic nucleotide assay methods highlights a significant advancement in analytical tools that has resolved previous uncertainties regarding the presence of cyclic nucleotides in plants. Today, the routine practice in the field of plant cyclic nucleotide research involves the application of multiple independent yet complementary methods, such as the complementation test for the *cyaA* mutation alongside detection techniques like LC-MS/MS or enzyme immunoassay. This comprehensive strategy not only bolsters evidence for the presence of cyclic nucleotides in plants but also sheds light on their roles in regulating various physiological processes, including growth, development, and stress responses. Overall, the evolution of cNMP assay methods represents a pivotal shift in plant biology, paving the way for future research that will further elucidate the complexities of cyclic nucleotide signaling.

## Computational approaches for analyzing plant cyclases and phosphodiesterases

Recognizing the crucial role of cyclic nucleotides in plant signaling, research efforts have concentrated on identifying the enzymes responsible for their synthesis, adenylyl cyclases and guanylyl cyclases, and degradation by phosphodiesterases. Advances in bioinformatics have been essential for uncovering these signaling pathways. Wong’s team reviewed strategies, workflows, and applications for developing search motifs specifically aimed at identifying AC, GC, and PDE activity in plant proteins (Wong et al., 2018; Zhou et al., 2021). A motif-based approach has been instrumental in identifying “functional centers” within multi-domain plant proteins, leading to the discovery of “moonlighting” or “crypto-GCs/ACs”, proteins with cyclase activity alongside their primary functions. This approach relies on conserved ‘functional center’ sequences, specialized peptide regions shorter than full domains or embedded within larger structures, which serve as dedicated functional sites. These motifs have proven effective in characterizing “crypto-cyclases,” enhancing our understanding of cyclase diversity in plants and offering new insights into multifunctional protein sites in plant signaling. The creation of conserved “functional center” sequences, defined as regions within a peptide sequence that are typically smaller than a regular domain and function as separate sites or are embedded within larger domains, has proven to be a powerful tool, especially in identifying plant cyclases functioning as crypto-cyclases. A 14-amino acid guanylyl cyclase search motif, denoted as [RKS][YFW][CTGH][VIL][FV]G[DNA]X[VIL]X{4}[KR][RKS], was initially derived from an alignment of catalytic regions across prokaryotes, lower eukaryotes, and higher eukaryotes (Ludidi and Gehring, 2003). Key functional residues are located in positions 1, 3, and 14 of this motif. Position 1 features amino acids [RKS], which form hydrogen bonds with the guanine base of the GTP substrate; position 3 [CTGH] imparts specificity for GTP; and position 14 [KR] stabilizes the transition state by binding to the phosphate acyl group as GTP is converted to cGMP. Using this GC motif, protein databases such as UniProt (http://www.uniprot.org) and organism-specific resources like TAIR (https://www.arabidopsis.org) were searched, leading to the discovery of the first active GC in *Arabidopsis thaliana* (AtGC1) (Gehring and Ludidi, 2003). This method has since identified additional GCs in other plant species, including *Hippeastrum hybridum* and *Brachypodium distachyon* (Kwezi et al., 2007; Meier et al., 2010; Qi et al., 2010; Muleya et al., 2014; Wheeler et al., 2017; Turek and Gehring, 2016; Świeżawska et al., 2017; Duszyn et al., 2021).

A similar motif-based approach was applied to adenylyl cyclase discovery. In 2010, Gehring introduced a 14-amino acid AC motif, [RK][YFW][DE][VIL][FV]X(8)[KR], aimed at identifying plant proteins with potential AC activity (Gehring, 2010). Searches using this AC motif have revealed numerous candidates containing the AC center in *A. thaliana* and *Zea mays* (Lemtiri-Chlieh et al., 2011), ultimately leading to the discovery of biochemically active plant adenylyl cyclases in subsequent studies (Al-Younis et al., 2015; 2018; Świeżawska et al., 2014). Based on the GC and AC search motifs, two predictive web tools, GCPred (http://gcpred.com) and ACPred (http://acpred.com), were developed by research groups under the supervision of Wong (Xu et al., 2018a, b). These tools enable the rapid prediction of candidate GC and AC centers, providing ranked results that help researchers identify hits with high confidence. This functionality streamlines the selection process, allowing researchers to focus on the most promising candidates for further study.

The identification and structural characterization of cyclic nucleotide phosphodiesterases, enzymes that degrade cNMPs, remained elusive in higher plants for many years. However, in 2021, researchers applied a combined motif-based and structural approach, creating a consensus sequence motif for PDEs embedded within complex multifunctional proteins, similar to plant cyclases (Kwiatkowski et al., 2021a, b). By aligning the catalytic centers of known PDEs from yeast and animals, they constructed a highly conserved motif: [YFW]Hx[YFW]Rx{20,40}[HRK][DE]. Using this motif to search the *Arabidopsis thaliana* proteome, they identified 32 potential PDE candidates, including two K^+^ transporters, AtKUP5 and AtKUP7, previously characterized as adenylyl cyclases with AC activity critical to their ion transport function (Al-Younis et al., 2015; 2018; 2021).

A similar dual-function AC-PDE structure was identified in the MpCAPE protein of the liverwort *Marchantia polymorpha*, suggesting an ancient signaling module where a single protein dynamically regulates localized cAMP levels (Kasahara et al., 2016). This dual functionality in such proteins could represent an evolutionary mechanism for finely tuned, transient control of cyclic nucleotide signaling in plants.

It’s important to emphasize that while bioinformatics approaches are invaluable in AC/GC/PDE research, they represent just the initial step. Searching protein databases using the described motifs—especially in their relaxed forms—often yields a large number of hits. However, the mere presence of such motifs does not constitute evidence of enzymatic activity. Many of these hits are likely to be false positives, highlighting the critical need for subsequent experimental validation to confirm activity of the potential AC/GC/PDE candidates. Only by combining computational techniques, such as motif prediction, structural analysis, and molecular docking, with rigorous experimental methods, including molecular biology and biochemical assays, can researchers achieve reliable and robust results.

## Milestones in cNMP research field in plants

**Figure 2:**
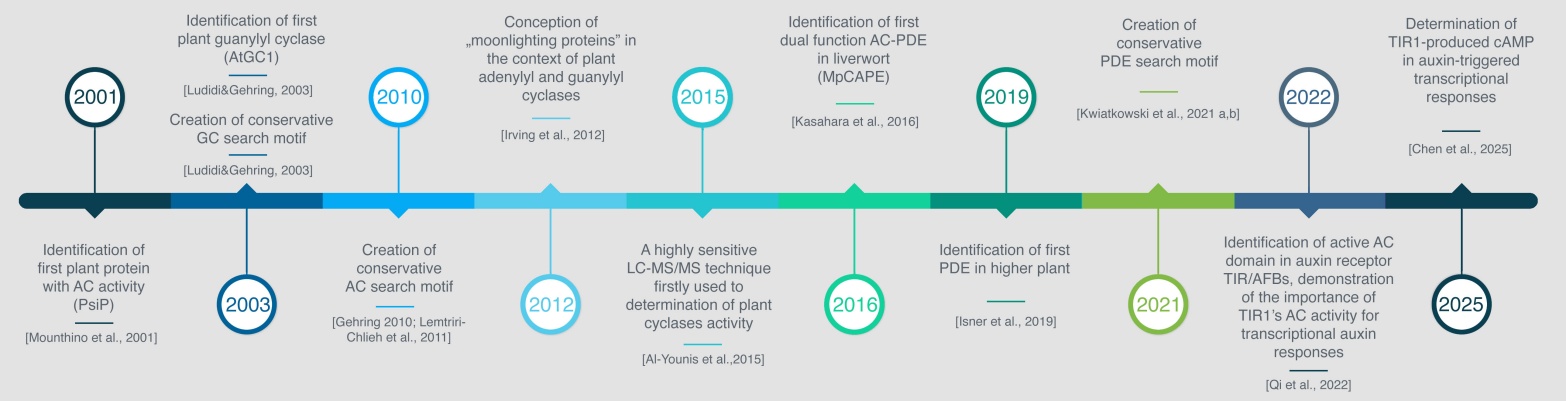
Timeline of advancements in plant cNMP research.

Establishing the current understanding of cyclic nucleotide regulating mechanisms in plants has been a long and intricate journey, marked by several key milestones (**Figure 2**). Among these, significant discoveries have paved the way for further research. The identification of plant cyclases, PsiP, the potential adenylyl cyclase in *Z. mays* (Moutinho et al., 2001), AtGC1, experimentally confirmed guanylyl cyclase in *A.thaliana* (Ludidi and Gehring, 2003) and AtCN-PDE1, the phosphodiesterase (Isner et al., 2019) set the stage for a wave of studies exploring the sequences, structures, biochemical properties, and functions of the enzymes responsible for cNMP synthesis and hydrolysis.

A pivotal breakthrough in the identification of novel GCs and ACs was the development of short, conserved search motifs consisting of 14 amino acids (Ludidi and Gehring, 2003; Gehring, 2010). Modifying and relaxing these motifs led to the discovery of several proteins containing hidden GC catalytic centers within their intracellular kinase domains. This protein architecture has been observed and tested in various receptors, including brassinosteroid insensitive 1 (BRI1) (Kwezi et al., 2007), phytosulfokine receptor (PSKR1) (Kwezi et al., 2011; Muleya et al., 2014), wall-associated kinase-like proteins (AtWAKL10, OsWAKL21.2) (Meier et al., 2010; Malukani et al., 2020), diacylglycerol kinases DGKs (Vaz Dias et al., 2019; Wong et al., 2020), plant natriuretic peptide receptor 1 (PNP-R1) (Turek and Gehring, 2016), extracellular ATP (eATP) receptors DORN1/P2K1 and P2K2 (Sun et al., 2021) and peptide signaling molecule receptors (AtPepR1, HpPepR1, BdPepR2, SlGC17, and SlGC18) (Qi et al., 2010; Świeżawska et al., 2017; Duszyn et al., 2021; Rahman et al., 2020). Notably, the coexistence of a kinase domain with an AC motif, but lacking a GC motif, was experimentally confirmed for the first time in the pentatricopeptide repeat protein (AtPPR) (Dikobe et al., 2024).

Interestingly, recent findings have indicated the presence of previously undiscovered functional “crypto-GCs” with conserved 14-amino acid-long motifs within the *Homo sapiens* proteome (Turek et al., 2023). The authors revealed that neurotrophic receptor tyrosine kinase 1 (NTRK1) shares a domain architecture similar to plant receptor kinases, such as PSKR1, with a functional GC embedded within a kinase domain essential for downstream signaling. This suggests that the phenomenon of hidden AC/GC motifs can be evident across multiple kingdoms of life (Turek et al., 2024).

In parallel, the development of conserved AC search motifs has proven to be an invaluable tool for predicting potential adenylyl cyclases, which have been subsequently validated through experimental work (Gehring, 2010). Hidden AC motifs have been identified in various multifunctional proteins across different plant species, including *A.thaliana*, *B. distachyon*, *Z. mays*, *Malus domestica*, *Brassica napus*, *Ziziphus jujuba*, and *H. hybridum*. Key examples include: two K^+^ transporters from *A. thaliana* (AtKUP5 and AtKUP7) (Al-Younis et al., 2015, 2018), clathrin assembly protein (AtClAP) (Chatukuta et al., 2018), leucine-rich repeat protein (AtLRRAC1) (Bianchet et al., 2019), 9-cis-epoxycarotenoid dioxygenase (NCED3) (Al-Younis et al., 2021), auxin receptors TIR1/AFB (Qi et al., 2022), putative disease-resistance protein RPP13-like (ZmRPP13-LK3) (Yang et al., 2021), and proteins belonging to the CYTH domain family (HpAC1, ZjAC, BdTTM3, MdTTM1, and MdTTM2) (Świeżawska et al., 2014, 2020; Liu et al., 2023; Yuan et al., 2022, 2023). A recent papers have provided a comprehensive overview of the discovery and roles of plant ACs in higher plants (Wong et al., 2023; Qi and Friml, 2023).

A transformative advancement in plant biochemistry has been the recognition of plant guanylyl cyclases as “moonlighting proteins” with dual catalytic functions, a concept introduced by Irving in 2012 (Irving et al., 2012). Initially applied to GCs, this paradigm has since expanded to include “crypto-ACs” (Al-Younis et al., 2021) and “hidden PDEs” (Kwiatkowski et al., 2021a, b), reshaping our understanding of cyclic nucleotide metabolism in plants. Rather than acting as isolated, single-function enzymes, these “cNMP-processing enzymes” are now understood to function as embedded, multifunctional domains within larger, complex proteins.

This reconceptualization led to groundbreaking discoveries, such as the identification of phosphodiesterase domains in MpCAPE, an enzyme capable of both synthesizing and hydrolyzing cAMP in the liverwort *Marchantia polymorpha*, and later in higher plants like *A. thaliana* and *Brassica napus* (Kasahara et al., 2016; Kwiatkowski et al., 2021a, b). The latest classification system defines adenylyl and guanylyl cyclases as generators of “on” signals, while phosphodiesterases act as generators of “off” signals within the plant cNMP signaling network (Kwiatkowski et al., 2024).

A recent breakthrough in plant signaling research was the identification of an adenylyl cyclase domain embedded within the TIR1/AFB auxin receptors, offering new insights into the complexity of phytohormone signaling (Qi et al., 2022). Building on this, a study published in *Nature* (Chen et al., 2025) has significantly reshaped our understanding of auxin signal transduction. The authors demonstrated that the AC activity of the TIR1 receptor is not just accessory but essential for triggering downstream transcriptional responses. Strikingly, they found that auxin-induced degradation of Aux/IAA repressors alone is insufficient to activate transcription. Instead, local cAMP production near the Aux/IAA–ARF complex, whether via unrelated ACs, can independently initiate ARF-mediated transcription. Recent findings have further expanded the complexity of auxin signaling by demonstrating that TIR1/AFB auxin receptors possess also guanylyl cyclase activity. Notably, in contrast to cAMP, cGMP has been shown to mediate rapid root responses, suggesting that auxin-induced cGMP production via TIR1/AFBs may play a role in triggering fast, non-transcriptional signaling events in auxin signaling pathways (Qi et al., 2023). These findings open exciting avenues for further exploration into whether these “on” and “off” signal generators (ACs, GCs and PDEs) play similar roles across other plant hormone pathways, raising important questions about the specificity and conservation of cyclic nucleotide signaling in plant biology. However, despite significant progress in the identification of plant proteins with biochemically confirmed AC/GC/PDE activity, a fully resolved and experimentally supported hormone signaling pathway involving cyclic nucleotides remains elusive. The field still lacks a comprehensive model that connects signal perception, nucleotide synthesis or hydrolysis, and the downstream action of cNMP effectors (e.g. cyclic nucleotide-gated channels, CNGCs, cAMP/cGMP-dependent protein kinases). Even in well-studied plant hormone pathway, such as auxin signaling, where the production of cAMP and cGMP has been demonstrated the complete mechanisms by which cNMPs potentially affect downstream effectors remain to be elucidated.

## Toward a new era of cyclic nucleotide research in plants

The study of cyclic nucleotides in plants is at an exciting juncture, poised to uncover new regulatory mechanisms that could reshape our understanding of plant biology. Looking forward, several promising directions hold potential for major advancements.

### Expanding the functional landscape of cNMPs in plants

As research begins to identify and validate more plant cyclases and phosphodiesterases, evidence is mounting that cAMP and cGMP play essential roles in various plant processes, from development and growth to stress responses and immune signaling. However, the specific downstream signaling pathways and cellular targets, such as protein kinases, ion channels, and transcription factors, remain largely unexplored. A deeper understanding of these downstream targets could illuminate new aspects of plant physiology and provide insights into how plants perceive and respond to environmental cues.

### Uncovering novel cNMP signaling pathways

Plants may also possess unique cNMP signaling pathways. Research is just beginning to uncover “moonlighting” enzymes, proteins that serve multiple functions. These multifunctional proteins, with hidden cyclase domains, suggest that cNMP signaling might be integrated into other critical pathways within plants. Future studies could reveal how these complex signaling networks interconnect, providing an intricate picture of cellular regulation in plants.

### Combining advanced bioinformatics and genetic tools to reveal the functional relevance of plant AC/GC/PDE

Computational tools have been instrumental in identifying and classifying potential cNMP-related enzymes in plants. The creation of predictive tools has allowed researchers to locate hidden cyclase and phosphodiesterase motifs within large, multifunctional proteins. However, to fully elucidate the functional relevance of AC, GC and PDE activities in plant signaling, future studies must go beyond biochemical characterization and investigate their roles *in planta* within specific signal transduction pathways. This includes the application of genetic approaches such as targeted knockouts, overexpression lines, and site-directed mutagenesis. Moreover, real-time imaging using genetically encoded cNMP biosensors provides a powerful strategy for tracking the spatial and temporal dynamics of cyclic nucleotide levels under various physiological conditions. Using of complementary structural biology techniques (e.g. X-ray crystallography) can also help elucidate conformational changes upon ligand binding or post-translational modifications. Ultimately, integrating these methodologies will be essential to establish the *in planta* functions of these enzymes and the signaling roles of their products.

### Deciphering cNMP roles in plant stress and immune responses

Evidence suggests that cyclic nucleotides may play a critical role in plant responses to biotic and abiotic stressors, such as pathogens, drought, and salinity. Exploring the details of cNMP involvement in stress tolerance and immune pathways could have practical implications for agriculture, where enhancing stress resilience in crops is a pressing need.

### Investigating cross-kingdom comparisons and evolutionary insights

Comparative analyses have revealed that cyclic nucleotide pathways share some conserved features across bacteria, fungi, plants, and animals. However, plant cNMP signaling pathways appear to exhibit unique adaptations that reflect evolutionary divergence. Exploring these differences can provide insights into the evolution of cell signaling mechanisms across life forms and reveal how plants have tailored cNMP pathways to meet their specific physiological needs.

The study of cyclic nucleotides in plants is progressing rapidly, yet much remains unknown. Future research will likely uncover novel enzymes, pathways, and regulatory mechanisms. These discoveries could transform our understanding of plant signaling and physiology, opening new avenues for agricultural innovation and providing insights into the evolutionary complexity of cellular communication. With ongoing advancements in bioinformatics, molecular biology, and biotechnology, plant cyclic nucleotide research promises to be a highly impactful field in the years to come.

## References

[B1] Świeżawska B. Duszyn M. Kwiatkowski M. Jaworski K. Pawełek A. Szmidt-Jaworska A. (2020). *Brachypodium distachyon* triphosphate tunnel metalloenzyme 3 is both a triphosphatase and an adenylyl cyclase upregulated by mechanical wounding. FEBS Lett. 594, 1101–1111. doi: 10.1002/1873-3468.13701 31785160

[B2] Świeżawska B. Jaworski K. Duszyn M. Pawełek A. Szmidt-Jaworska A. (2017). The *Hippeastrum hybridum* PepR1 gene (*HpPepR1*) encodes a functional guanylyl cyclase and is involved in early response to fungal infection. J.Plant Physiol. 216, 100–107. doi: 10.1016/j.jplph.2017.05.024 28609666

[B3] Świeżawska B. Jaworski K. Pawełek A. Grzegorzewska W. Szewczuk P. Szmidt-Jaworska A. (2014). Molecular cloning and characterization of a novel adenylyl cyclase gene, *HpAC1*, involved in stress signaling in *Hippeastrum × hybridum* . Plant Physiol. Biochem. 80, 41–52. doi: 10.1016/j.plaphy.2014.03.018 24721550

[B4] Świeżawska B. Jaworski K. Szewczuk P. Pawełek A. Szmidt-Jaworska A. (2015). Identification of a *Hippeastrum hybridum* guanylyl cyclase responsive to wounding and pathogen infection. J. Plant Physiol. 189, 77–86. doi: 10.1016/j.jplph.2015.09.014 26523507

[B5] Al-Younis I. Moosa B. Kwiatkowski M. Jaworski K. Wong A. Gehring C. (2021). Functional crypto-adenylate cyclases operate in complex plant proteins. Front. Plant Sci. 12. doi: 10.3389/fpls.2021.711749 PMC8387589 34456950

[B6] Al-Younis I. Wong A. Gehring C. (2015). The *Arabidopsis thaliana* K⁺-uptake permease 7 (AtKUP7) contains a functional cytosolic adenylate cyclase catalytic centre. FEBS Lett. 589, 3848–3852. doi: 10.1016/j.febslet.2015.11.038 26638082

[B7] Al-Younis I. Wong A. Lemtiri-Chlieh F. Schmöckel S. Tester M. Gehring C.. (2018). The *Arabidopsis thaliana* K⁺-Uptake Permease 5 (AtKUP5) contains a functional cytosolic adenylate cyclase essential for K⁺ transport. Front. Plant Sci. 9. doi: 10.3389/fpls.2018.01645 PMC6243130 30483296

[B8] Amrhein N. (1974). Evidence against the occurrence of adenosine-3′:5′-cyclic monophosphate in higher plants. Planta 118, 241–258. doi: 10.1007/BF00384780 24442328

[B9] Ashton A. R. (2011). Guanylyl cyclase activity in plants? Proc. Natl. Acad. Sci. U.S.A. 108, E96. doi: 10.1073/pnas.1101007108 21527716 PMC3093516

[B10] Assmann S. M. (1995). Cyclic AMP as a second messenger in higher plants (status and future prospects). Plant Physiol. 108, 885–889. doi: 10.1104/pp.108.3.885 12228514 PMC157436

[B11] Azhar S. Murti C. R. (1971). Effect of indole-3-acetic acid on the synthesis of cyclic 3′,5′ adenosine phosphate by Bengal gram seeds. Biochem. Biophys. Res. Commun. 43, 58–64. doi: 10.1016/s0006-291x(71)80085-2 4325495

[B12] Beavo J. A. Brunton L. L. (2002). Cyclic nucleotide research – still expanding after half a century. Nat. Rev. Mol. Cell Biol. 3, 710–718. doi: 10.1038/nrm911 12209131

[B13] Berkowitz G. A. Gehring C. Irving H. R. Kwezi L. (2011). Reply to Ashton: The putative guanylyl cyclase domain of AtPepR1 and similar plant receptors. Proc. Natl. Acad. Sci. U.S.A. 108, E97–E98. doi: 10.1073/pnas.1103313108

[B14] Bianchet C. Wong A. Quaglia M. Alqurashi M. Gehring C. Ntoukakis V.. (2019). *Arabidopsis thaliana* leucine-rich repeat protein harbors an adenylyl cyclase catalytic center and affects responses to pathogens. J. Plant Physiol. 232, 12–22. doi: 10.1016/j.jplph.2018.10.025 30530199

[B15] Blanco E. Fortunato S. Viggiano L. de Pinto M. C. (2020). Cyclic AMP: a polyhedral signalling molecule in plants. Int. J. Mol. Sci. 21, 4862. doi: 10.3390/ijms21144862 32660128 PMC7402341

[B16] Bojar D. Martinez J. Santiago J. Rybin V. Bayliss R. Hothorn M. (2014). Crystal structures of the phosphorylated BRI1 kinase domain and implications for brassinosteroid signal initiation. Plant J. 78, 31–43. doi: 10.1111/tpj.12445 24461462 PMC4260089

[B17] Bolwell G. P. (1992). A role for phosphorylation in the down-regulation of phenylalanine ammonia-lyase in suspension-cultured cells of *Phaseolus vulgaris* . Phytochemistry 31, 4081–4086. doi: 10.1016/0031-9422(92)80418-E

[B18] Brooker G. (1982). “Radioimmunoassay techniques for cyclic nucleotides,” in *Cyclic nucleotides. Handb. Exp. Pharmacol* , vol. 58 . Eds. Nathanson J. A. Kebabian J. W. (Springer, Berlin, Heidelberg), 193–207. doi: 10.1007/978-3-642-68111-0_8

[B19] Brown E. G. Newton R. P. (1973). Occurrence of adenosine 3′,5′-cyclic monophosphate in plant tissues. Phytochemistry 12, 2683–2685. doi: 10.1016/0031-9422(73)85080-0

[B20] Brown E. G. Newton R. P. (1981). Cyclic AMP and higher plants. Phytochemistry 20, 2453–2463. doi: 10.1016/0031-9422(81)83071-3

[B21] Chatukuta P. Dikobe T. B. Kawadza D. T. Sehlabane K. S. Takundwa M. M. Wong A.. (2018). An *Arabidopsis* clathrin assembly protein with a predicted role in plant defense can function as an adenylate cyclase. Biomolecules 8, 15. doi: 10.3390/biom8010015 29570675 PMC6022867

[B22] Chen H. Qi L. Zou M. Lu M. Kwiatkowski M. Pei Y.. (2025). TIR1-produced cAMP as a second messenger in transcriptional auxin signalling. Nature 640, 1011–1016. doi: 10.1038/s41586-025-08669-w 40044868 PMC12018254

[B23] Cousson A. Vavasseur A. (1998). Putative involvement of cytosolic Ca2+ and GTP-binding proteins in cyclic-GMP-mediated induction of stomatal opening by auxin in Commelina communis L. Planta 206, 308–314. doi: 10.1007/s004250050405

[B24] Dikobe T. Sehlabane K. Bobo E. Sibanda-Makuvise A. Chatukuta P. Kawadza D.. (2024). An *Arabidopsis* pentatricopeptide repeat is a moonlighting protein with cross-talking *in vitro* adenylyl cyclase and kinase activities. Plant Mol. Biol. Rep. 42, 77–88. doi: 10.1007/s11105-023-01401-w

[B25] Duszyn M. Świeżawska-Boniecka B. Wong A. Jaworski K. Szmidt-Jaworska A. (2021). *In vitro* characterization of guanylyl cyclase BdPepR2 from *Brachypodium distachyon* identified through a motif-based approach. Int. J. Mol. Sci. 22, 6243. doi: 10.3390/ijms22126243 34200573 PMC8228174

[B26] Figueroa N. E. Franz P. Luzarowski M. Martinez-Seidel F. Moreno J. C. Childs D.. (2023). Protein interactome of 3′,5′-cAMP reveals its role in regulating the actin cytoskeleton. Plant J. 115, 1214–1230. doi: 10.1111/tpj.16313 37219088

[B27] Gehring C. (2010). Adenyl cyclases and cAMP in plant signaling – past and present. Cell Commun. Signal. 8, 15. doi: 10.1186/1478-811X-8-15 20579354 PMC2907374

[B28] Gilman A. G. (1970). A protein binding assay for adenosine 3′,5′-cyclic monophosphate. Proc. Natl. Acad. Sci. U.S.A. 67, 305–312. doi: 10.1073/pnas.67.1.305 4318781 PMC283204

[B29] Harper J. F. Brooker G. (1975). Femtomole sensitive radioimmunoassay for cyclic AMP and cyclic GMP after 2′/0 acetylation by acetic anhydride in aqueous solution. J. Cyclic Nucl. Res. 1, 207–218. 177461

[B30] Ichikawa T. Suzuki Y. Czaja I. Schommer C. Lessnick A. Schell J (1998). Identification and role of adenylyl cyclase in auxin signalling in higher plants. Nature 396–390. doi: 10.1038/24659 9845078

[B31] Irving H. R. Gehring C. Wong A. (2025). Cryptic enzymes and moonlighting proteins (Amsterdam: Elsevier).

[B32] Irving H. R. Kwezi L. Wheeler J. Gehring C. (2012). Moonlighting kinases with guanylate cyclase activity can tune regulatory signal networks. Plant Signal. Behav. 7, 201–204. doi: 10.4161/psb.18891 22353864 PMC3405710

[B33] Ishioka N. Tanimoto S. (1990). Involvement of cyclic AMP in adventitious bud initiation of *Torenia* stem segments. Plant Cell Physiol. 31, 91–97. doi: 10.1093/oxfordjournals.pcp.a077886

[B34] Isner J. C. Nühse T. Maathuis F. J. M. (2012). The cyclic nucleotide cGMP is involved in plant hormone signalling and alters phosphorylation of Arabidopsis thaliana root proteins. J. Exp. Bot. 63, 3199–3205. doi: 10.1093/jxb/ers045 22345640 PMC3350932

[B35] Isner J. C. Olteanu V. A. Hetherington A. J. Coupel-Ledru A. Sun P. Pridgeon A. J.. (2019). Short- and long-term effects of UVA on Arabidopsis are mediated by a novel cGMP phosphodiesterase. Curr. Biol. 29, 2580–2585.e4. doi: 10.1016/j.cub.2019.06.071 31353185 PMC6692503

[B36] Janistyn B. (1972). 3-Indole acetic-acid induced efflux of nucleotides concomitant with an increased synthesis of adenosine-3′,5′-monophosphate (cAMP) in maize coleoptile sections. Z. für Naturforschung B 27, 273–276. doi: 10.1515/znb-1972-0310 4402628

[B37] Jin X. C. Wu W. H. (1999). Involvement of cyclic AMP in ABA- and Ca2+-mediated signal transduction of stomatal regulation in Vicia faba. Plant Cell Physiol. 40, 1127–1133. doi: 10.1093/oxfordjournals.pcp.a029497

[B38] Johnson L. P. MacLeod J. K. Parker C. W. Letham D. S. Hunt N. H. (1981). Identification and quantitation of adenosine-3′:5′-cyclic monophosphate in plants using gas chromatography–mass spectrometry and high-performance liquid chromatography. Planta 152, 195–201. doi: 10.1007/BF00385144 24302415

[B39] Kasahara M. Suetsugu N. Urano Y. Yamamoto C. Ohmori M. Takada Y. (2016). An adenylyl cyclase with a phosphodiesterase domain in basal plants with a motile sperm system. Sci. Rep. 6, 39232. doi: 10.1038/srep39232 27982074 PMC5159850

[B40] Keates R. A. B. (1973). Evidence that cyclic-AMP does not mediate action of gibberellic acid. Nature 244, 355–357. doi: 10.1038/244355a0

[B41] Kleinboelting S. Miehling J. Steegborn C. (2020). Crystal structure and enzymatic characterization of the putative adenylyl cyclase HpAC1 from *Hippeastrum* reveal dominant triphosphatase activity. J. Struct. Biol. 212, 107649. doi: 10.1016/j.jsb.2020.107649 33075486

[B42] Kwezi L. Meier S. Mungur L. Ruzvidzo O. Irving H. Gehring C. (2007). The *Arabidopsis thaliana* brassinosteroid receptor (AtBRI1) contains a domain that functions as a guanylyl cyclase *in vitro* . PloS One 2, e449. doi: 10.1371/journal.pone.0000449 17520012 PMC1867859

[B43] Kwezi L. Ruzvidzo O. Wheeler J. I. Govender K. Iacuone S. Thompson P. E.. (2011). The phytosulfokine (PSK) receptor is capable of guanylate cyclase activity and enabling cyclic GMP-dependent signaling in plants. J. Biol. Chem. 286, 22580–22588. doi: 10.1074/jbc.M110.168823 21504901 PMC3121402

[B44] Kwiatkowski M. Wong A. Fiderewicz A. Gehring C. Jaworski K. (2024). A SNF1-related protein kinase regulatory subunit functions as a molecular tuner. Phytochem. 224, 114–146. doi: 10.1016/j.phytochem.2024.114146 38763313

[B45] Kwiatkowski M. Wong A. Kozakiewicz A. Gehring C. Jaworski K. (2021a). A tandem motif-based and structural approach can identify hidden functional phosphodiesterases. Comput. Struct. Biotechnol. J. 19, 970–975. doi: 10.1016/j.csbj.2021.01.036 33613864 PMC7873575

[B46] Kwiatkowski M. Wong A. Kozakiewicz-Piekarz A. Gehring C. Jaworski K. (2021b). In search of monocot phosphodiesterases: identification of a calmodulin stimulated phosphodiesterase from *Brachypodium distachyon* . Int. J. Mol. Sci. 22, p.9654. doi: 10.3390/ijms22179654 PMC8431786 34502563

[B47] Lefkimmiatis K. Moyer M. P. Curci S. Hofer A. M. (2009). cAMP sponge”: a buffer for cyclic adenosine 3′,5′-monophosphate. PloS One 4, e7649. doi: 10.1371/journal.pone.0007649 19888343 PMC2766031

[B48] Lemtiri-Chlieh F. Thomas L. Marondedze C. Irving H. Gehring C. (2011). “Cyclic nucleotides and nucleotide cyclases in plant stress responses,” in Abiotic stress in plants – mechanisms and adaptations. Ed. Shanker A. (InTech, Rijeka). doi: 10.5772/24757

[B49] Liu Z. Zhang H. Wang P. Zhao X. Wang L. Wang L.. (2023). Three novel adenylate cyclase genes show significant biological functions in plant. J. Agric. Food Chem. 71, 1149–1161. doi: 10.1021/acs.jafc.2c07991 36601683

[B50] Ludidi N Gehring C. (2003) Identification of a novel protein with guanylyl cyclase activity in Arabidopsis thaliana. J Biol Chem. 278(8), 6490–4. doi: 10.1074/jbc.M210983200 12482758

[B51] Malukani K. K. Ranjan A. Hota S. J. Patel H. K. Sonti R. V. (2020). Dual activities of receptor-like kinase OsWAKL21.2 induce immune responses. Plant Phys. 183, 1345–1363. doi: 10.1104/pp.19.01579 PMC7333719 32354878

[B52] Meier S. Ruzvidzo O. Morse M. Donaldson L. Kwezi L. Gehring C. (2010). The *Arabidopsis* wall associated kinase-like 10 gene encodes a functional guanylyl cyclase and is co-expressed with pathogen defense related genes. PloS One 5, e8904. doi: 10.1371/journal.pone.0008904 20126659 PMC2811198

[B53] Moutinho A. Hussey P. J. Trewavas A. J. Malhó R. (2001). cAMP acts as a second messenger in pollen tube growth and reorientation. Proc. Natl. Acad. Sci. U.S.A. 98, 10481–10486. doi: 10.1073/pnas.171104598 11517303 PMC56986

[B54] Muleya V. Wheeler J. I. Ruzvidzo O. Freihat L. Manallack D. T. Gehring C. (2014). Calcium is the switch in the moonlighting dual function of the ligand-activated receptor kinase phytosulfokine receptor 1. Cell Commun. Signal 12, 60. doi: 10.1186/s12964-014-0060-z 25245092 PMC4180545

[B55] Penson S. P. Schuurink R. C. Fath A. Gubler F. Jacobsen J. V. Jones R. L. (1996). cGMP is required for gibberellic acid-induced gene expression in barley aleurone. Plant Cell 8, 2325–2333. doi: 10.1105/tpc.8.12.2325 12239379 PMC161355

[B56] Qi L. Friml J. (2023). Tale of cAMP as a second messenger in auxin signaling and beyond. New Phytol. 240, 489–495. doi: 10.1111/nph.19123 37434303 PMC10952583

[B57] Qi L. Kwiatkowski M. Chen H. Hoermayer L. Sinclair S. Zou M.. (2022). Adenylate cyclase activity of TIR1/AFB auxin receptors in plants. Nature 611, 133–138. doi: 10.1038/s41586-022-05369-7 36289340

[B58] Qi L. Kwiatkowski M. Kulich I. Chen H. Gao Y. Yun P.. (2023). Guanylate cyclase activity of TIR1/AFB auxin receptors in rapid auxin responses. bioRxiv. doi: 10.1101/2023.11.18.567481

[B59] Qi Z. Verma R. Gehring C. Yamaguchi Y. Zhao Y. Ryan C. A.. (2010). Ca²⁺ signaling by plant *Arabidopsis thaliana* Pep peptides depends on AtPepR1, a receptor with guanylyl cyclase activity, and cGMP-activated Ca²⁺ channels. Proc. Natl. Acad. Sci. U.S.A. 107, 21193–21198. doi: 10.1073/pnas.1000191107 21088220 PMC3000296

[B60] Rahman H. Wang X. Y. Xu Y. P. He Y. H. Cai X. Z. (2020). Characterization of tomato protein kinases embedding guanylate cyclase catalytic center motif. Sci.Rep. 10, 4078. doi: 10.1038/s41598-020-61000-7 32139792 PMC7057975

[B61] Ruzvidzo O. Gehring C. Wong A. (2019). New perspectives on plant adenylyl cyclases. Front. Mol. Biosci. 6. doi: 10.3389/fmolb.2019.00136 PMC6901789 31850369

[B62] Sabetta W. Vandelle E. Locato V. Costa A. Cimini S. Bittencourt Moura A.. (2019). Genetic buffering of cyclic AMP in *Arabidopsis thaliana* compromises the plant immune response triggered by an avirulent strain of *Pseudomonas syringae* pv. tomato. Plant J. 98, 590–606. doi: 10.1111/tpj.14275 30735606

[B63] Sabetta W. Vannini C. Sgobba A. Marsoni M. Paradiso A. Ortolani F.. (2016). Cyclic AMP deficiency negatively affects cell growth and enhances stress-related responses in tobacco Bright Yellow-2 cells. Plant Mol. Biol. 90, 467–483. doi: 10.1007/s11103-016-0431-5 26786166

[B64] Salomon D. Mascarenhas J. P. (1972). Time course of synthesis of cyclic AMP in *Avena* coleoptile sections in response to auxin. Plant Physiol. 49, 30.

[B65] Shah S. Peterkofsky A. (1991). Characterization and generation of *Escherichia coli* adenylate cyclase deletion mutants. J. Bacteriol. 173, 3238–3242. doi: 10.1128/jb.173.10.3238-3242.1991 2022622 PMC207922

[B66] Spiteri A. Viratelle O. M. Raymond P. Rancillac M. Labouesse J. Pradet A. (1989). Artefactual origins of cyclic AMP in higher plant tissues. Plant Physiol. 91, 624–628. doi: 10.1104/pp.91.2.624 16667078 PMC1062046

[B67] Straube H. Witte C. P. Herde M. (2021). Analysis of nucleosides and nucleotides in plants: an update on sample preparation and LC–MS techniques. Cells 10, 689. doi: 10.3390/cells10030689 33804650 PMC8003640

[B68] Sun J. Ning Y. Wang L. Wilkins K. A. Davies J. M. (2021). Damage signaling by extracellular nucleotides: A role for cyclic nucleotides in elevating cytosolic free calcium? Front. Plant Sci. 12. doi: 10.3389/fpls.2021.788514 PMC8675005 34925428

[B69] Sunahara R. K. Taussig R. (2002). Isoforms of mammalian adenylyl cyclase: multiplicities of signaling. Mol. Interv. 2, 168–184. doi: 10.1124/mi.2.3.168 14993377

[B70] Sutherland E. W. Rall T. W. (1957). The properties of an adenine ribonucleotide produced with cellular particles, ATP, Mg++, and epinephrine or glucagon. J. Am. Chem. Soc 79, 3608–3610. doi: 10.1021/ja01571a063

[B71] Świeżawska-Boniecka B Szmidt-Jaworska A. (2023). Phytohormones and cyclic nucleotides - Long-awaited couples? J Plant Physiol. 286, 154005. doi: 10.1016/j.jplph.2023s 37186984

[B72] Turek I. Freihat L. Vyas J. Wheeler J. Muleya V. Manallack D. T.. (2023). The discovery of hidden guanylate cyclases (GCs) in the *Homo sapiens* proteome. Comput. Struct. Biotechnol. J. 21, 5523–5529. doi: 10.1016/j.csbj.2023.11.005 38022692 PMC10665587

[B73] Turek I. Gehring C. (2016). The plant natriuretic peptide receptor is a guanylyl cyclase and enables cGMP-dependent signaling. Plant Mol. Biol. 91, 275–286. doi: 10.1007/s11103-016-0465-8 26945740

[B74] Turek I. Irving H. (2021). Moonlighting proteins shine new light on molecular signaling niches. Int. J. Mol. Sci. 22, p.1367. doi: 10.3390/ijms22031367 PMC7866414 33573037

[B75] Turek I. Wong A. Domingo G. Vannini C. Bracale M. Irving H.. (2024). Moonlighting crypto-enzymes and domains as ancient and versatile signaling devices. Int. J. Mol. Sci. 25, 9535. doi: 10.3390/ijms25179535 39273482 PMC11394779

[B76] Vaz Dias F. Serrazina S. Vitorino M. Marchese D. Heilmann I. Godinho M (2019). A role for diacylglycerol kinase 4 in signalling crosstalk during Arabidopsis pollen tube growth. New Phytol. 222, 1434–1446. doi: 10.1111/nph.15674 30628082

[B77] Visweswariah S. S. Jaiswal N. (2016). “Guanylyl cyclase receptors,” in Encyclopedia of signaling molecules. Ed. Choi S. (Springer, New York, NY). doi: 10.1007/978-1-4614-6438-9_434-1

[B78] Wheeler J. I. Wong A. Marondedze C. Groen A. J. Kwezi L. Freihat L. (2017). The brassinosteroid receptor BRI1 can generate cGMP enabling cGMP-dependent downstream signaling. Plant J. 91, 590–600. doi: 10.1111/tpj.13591 28482142

[B79] Witters E. Roef L. Newton R. P. Van Dongen W. Esmans E. L. Van Onckelen H. A. (1996). Quantitation of cyclic nucleotides in biological samples by negative electrospray tandem mass spectrometry coupled to ion suppression liquid chromatography. Rapid Commun. Mass Spectrom 10, 10225–10231. doi: 10.1002/(SICI)1097-0231(19960131)10:2

[B80] Witters E. Vanhoutte K. Dewitte W. Machackova I. Benkova E. Van Dongen W.. (1999). Analysis of cyclic nucleotides and cytokinins in minute plant samples using phase-system switching capillary electrospray-liquid chromatography-tandem mass spectrometry. Phytochem. Anal. 10, 143–151. doi: 10.1002/(SICI)1099-1565(199905/06)10:3

[B81] Wong A. Chi W. Yu J. Bi C. Tian X. Yang Y.. (2023). Plant adenylate cyclases have come full circle. Nat. Plants 9, 1389–1397. doi: 10.1038/s41477-023-01486-x 37709954

[B82] Wong A. Donaldson L. Portes M. T. Eppinger J. Feijó J. A. Gehring C. (2020). *Arabidopsis* DIACYLGLYCEROL KINASE4 is involved in nitric oxide-dependent pollen tube guidance and fertilization. Development 147, dev183715. doi: 10.1242/dev.183715 32220864 PMC13110872

[B83] Wong A. Tian X. Gehring C. Marondedze C. (2018). Discovery of novel functional centers with rationally designed amino acid motifs. Comput. Struct. Biotechnol. J. 16, 70–76. doi: 10.1016/j.csbj.2018.02.007 29977479 PMC6026216

[B84] Xu N. Fu D. Li S. Wang Y. Wong A. (2018a). GCPred: a web tool for guanylyl cyclase functional centre prediction from amino acid sequence. Bioinformatics 34, 2134–2135. doi: 10.1093/bioinformatics/bty067 29420675

[B85] Xu N. Zhang C. Lim L. L. Wong A. (2018b). “Bioinformatic Analysis of Nucleotide Cyclase Functional Centers and Development of ACPred Webserver,” (2018), (Kean Publications). 1475. Available online at:https://digitalcommons.kean.edu/keanpublications/1475

[B86] Yang H. Zhao Y. Chen N. Liu Y. Yang S. Du H.. (2021). A new adenylyl cyclase, putative disease-resistance RPP13-like protein 3, participates in abscisic acid-mediated resistance to heat stress in maize. J. Exp. Bot. 72, 283–301. doi: 10.1093/jxb/eraa431 32936902

[B87] Yuan Y. Liu Y. Chen S. Wang L. Wang L. Niu Y.. (2023). A triphosphate tunnel metalloenzyme from pear (PbrTTM1) moonlights as an adenylate cyclase. Front. Plant Sci. 14. doi: 10.3389/fpls.2023.1183931 PMC10324617 37426988

[B88] Yuan Y. Liu Z. Wang L. Wang L. Chen S. Niu Y.. (2022). Two triphosphate tunnel metalloenzymes from apple exhibit adenylyl cyclase activity. Front. Plant Sci. 13. doi: 10.3389/fpls.2022.992488 PMC9582125 36275530

[B89] Zhou W. Chi W. Shen W. Dou W. Wang J. Tian X.. (2021). Computational identification of functional centers in complex proteins: a step-by-step guide with examples. Front. Bioinform. 1. doi: 10.3389/fbinf.2021.652286 PMC9581015 36303732

